# Trauma care and capture rate of variables of World Health Organisation data set for injury at regional hospitals in Tanzania: first steps to a national trauma registry

**DOI:** 10.1186/s12873-020-00325-y

**Published:** 2020-04-23

**Authors:** Hendry R. Sawe, Teri A. Reynolds, Ellen J. Weber, Juma A. Mfinanga, Timothy J. Coats, Lee A. Wallis

**Affiliations:** 1 Department of Emergency Medicine, Muhimbili University of Health and Allied Sciences, P.O. Box 65001, Dar es Salaam, Tanzania; 2 Division of Emergency Medicine, Faculty of Health Sciences, University of Cape Town, Private Bag X24 • Bellville, Cape Town, 7535 South Africa; 3 Department for the Management of NCDs, Disability, Violence and Injury Prevention, Integrated Health Services, World Health Organization (WHO), 20, Avenue Appia, 1211 Geneva, Switzerland; 4 Emergency Department, University of California San-Francisco, 505 Parnassus Ave, San Francisco, CA 94143 USA; 5 Department of Emergency Medicine, Muhimbili National Hospital, P.O. Box 65001, Dar es Salaam, Tanzania; 6 Department of Cardiovascular Sciences, University of Leicester, University Road, Leicester, LE1 7RH UK

**Keywords:** Injury registry, Emergency care, Trauma burden, Trauma care, Africa, Tanzania

## Abstract

**Background:**

In Tanzania, there is no national trauma registry. The World Health Organization (WHO) has developed a data set for injury that specifies the variables necessary for documenting the burden of injury and patient-related clinical processes. As a first step in developing and implementing a national Trauma Registry, we determined how well hospitals currently capture the variables that are specified in the WHO injury set.

**Methods:**

This was a prospective, observational cross-sectional study of all trauma patients conducted in the Emergency Units of five regional referral hospitals in Tanzania from February 2018 to July 2018. Research assistants observed the provision of clinical care in the EU for all patients, and documented performed assessment, clinical interventions and final disposition. Research assistants used a purposefully designed case report form to audit the injury variable capture rate, and to review Ministry of Health (MoH) issued facility Register book recording the documentation of variables. We present descriptive statistics for hospital characteristics, patient volume, facility infrastructure, and capture rate of trauma variables.

**Results:**

During the study period, 2891 (9.3%) patients presented with trauma-related complaints, 70.7% were male. Overall, the capture rate of all variables was 33.6%. Documentation was most complete for demographics 71.6%, while initial clinical condition, and details of injury were documented in 20.5 and 20.8% respectively. There was no documentation for the care prior to Emergency Unit arrival in all hospitals. 1430 (49.5%) of all trauma-related visits seen were documented in the facility Health Management Information System register submitted to the MoH. Among the cases reported in the register book, the date of EU care was correctly documented in 77% cases, age 43.6%, diagnosis 66.7%, and outcome in 38.9% cases. Among the observed procedures, initial clinical condition (28.7%), interventions at Emergency Unit (52.1%), investigations (49.0%), and disposition (62.9%) were documented in the clinical charts.

**Conclusions:**

In the regional hospitals of Tanzania, there is inadequate documentation of the minimum trauma variables specified in the WHO injury data set. Reasons for this are unclear, but will need to be addressed in order to improve documentation to inform a national injury registry.

## Background

A trauma registry (TR) is a key infrastructural component of a well-functioning trauma care system, facilitating appropriate deployment of resources, quality improvement, injury prevention initiatives, injury-research execution, and policy development [1]. In most high income countries (HICs), parallel deployment of TR with development of formal trauma care systems has played a key role in decreasing injury-related disability and mortality [2]. Few low- and middle-income countries (LMICs) have TRs; in the places where they exist, they are poorly developed and managed, and in many cases unsustainable [3]. Trauma data are mostly reliant on single site, hospital-based chart reviews or mortuary-based data logs [4–7] which are often incomplete, or subject to interpretation.

In Tanzania, like most LMICs, there is a substantial shortage of both human and infrastructural resource for optimal provision of health services [8, 9]. The public health system, which attends to majority of patients, is organised in a pyramidal structure with most emergency care for trauma occurring at the lower tier of the system - most of which have no dedicated emergency care providers [8, 10]. Emergency Medicine is still a new concept in most of the regional hospitals, which are not well equipped and staffed to provide trauma care.

There is no national TR in Tanzania, and most of the trauma data that are published in the literature are based on isolated hospital-based initiatives [11–13]. Implementation of an appropriate model of TR in Tanzania faces several challenges, including the lack of standardized and uniform hospital records, lack of electronic charting for nearly all the hospitals, and inadequate health care system funding and staffing which makes it hard for the health facilities to designate personnel to compile TR data. The lack of TR has substantially affected the country’s ability to create initiatives to improve injury related outcomes, and plan for appropriate preventive measures against injuries.

The World Health Organization (WHO) has developed a data set for injury (DSI) that specifies the variables necessary for documenting the burden of injury and clinical processes, with the main goal of informing both quality improvement and system planning activities at the facility and at national level [14]. The WHO DSI has a set of core and extended variables that are categorized into: patients’ demographics, initial clinical condition, details of injury, pre-hospital and prior facility care, injury examination details, and Emergency Unit (EU) details, and in-patient details. An email (*WHO, Emergency Care Systems, Clinical Services and Systems Unit, personal communication, 16 March 2020*) confirmed that, WHO expects to publish the metadata for the WHO DSI as an open source, and at the moment member countries can request to participate in implementing WHO DSI, as well as utilizing the WHO International Registry for Trauma and Emergency Care for aggregation and analysis of injury case based data from emergency units [15]. The DSI has a component that will also allow patients details to be recorded. This data set was developed from country-specific TRs, with the advice of injury experts from across the world through a consensus process.

We propose to develop a TR for Tanzania based on the WHO DSI. As a first step in developing the TR, this study aimed to determine the current capture rate of trauma variables that form part of the WHO DSI. The results of this study will help to understand which of the variables are currently being documented, and therefore to what extent current trauma data are inadequate. This will provide the guide towards next steps in training of health care providers and availability of resources to develop a robust and sustainable TR model for Tanzania and, by extension, and other LMICs.

## Methods

### Study design

This was a prospective, observational cross-sectional study of all trauma patients (adult and paediatric) presenting to EU) at a sample of regional hospitals in Tanzania conducted for a period of six months, from 1st February 2018 and 31st July 2018.

### Study setting

**Fig. 1:**
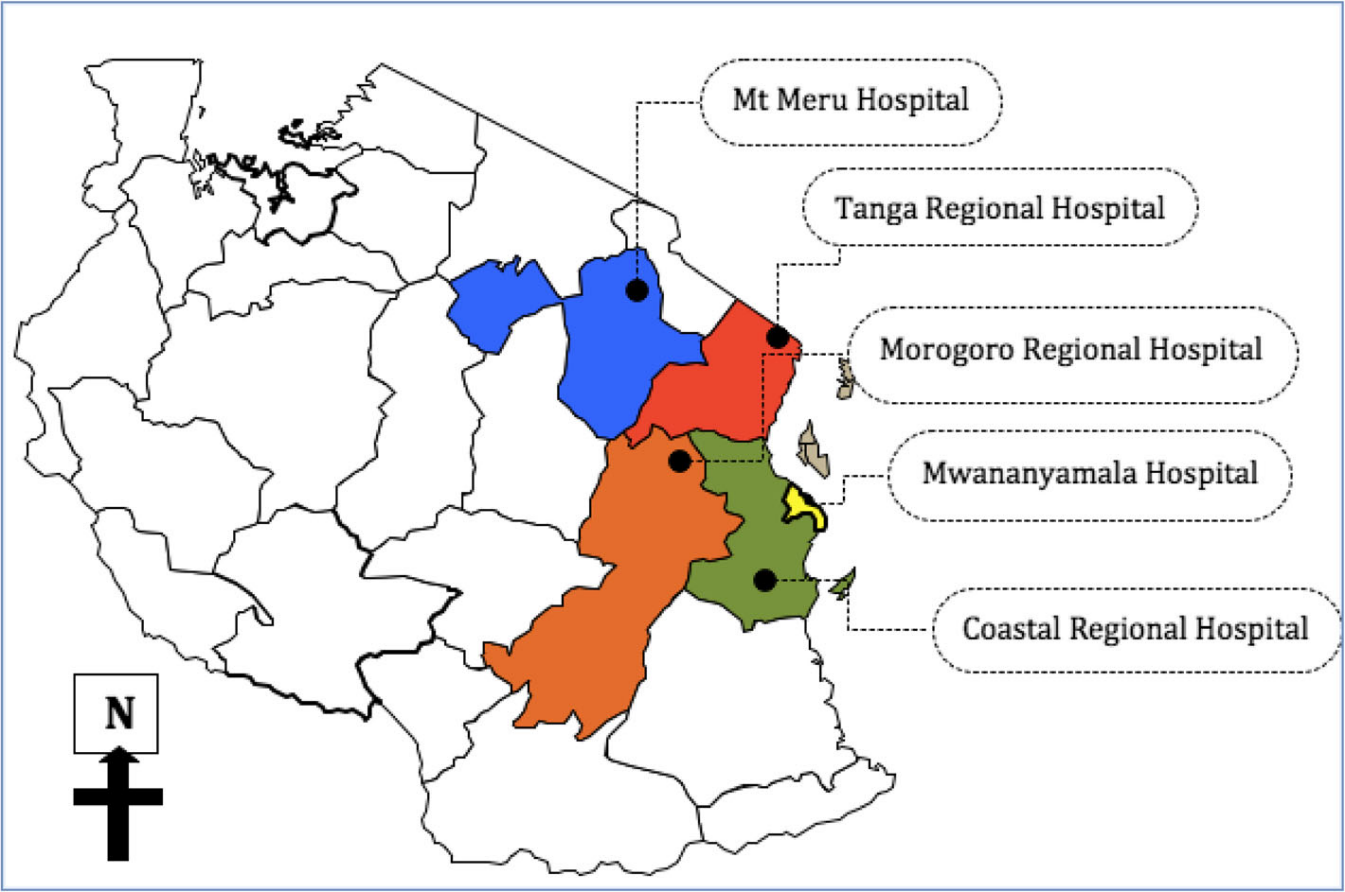
Map of Tanzania indicating study sites

The United Republic of Tanzania is a country with a population of 55 million people, located in the Eastern Africa, and designated as a Low Income Country with estimated per capita income of approximately US$ 920 [16]. At the time of the study, Tanzania had 25 geopolitical regions, and a public health system provided in a pyramidal structure from dispensary, health centre, district hospital and regional hospital to consultant hospitals. There is no formal trauma care system, hence trauma patients are taken to the nearest available health facility that might provide definitive care or refer the patient to a higher level of care, depending on the capacity and resource availability [17]. Most of the lower level facilities are not adequately prepared for stabilization of trauma patients needing emergency care [18, 19]. This study was conducted in the EU of five regional referral hospitals in Tanzania: Morogoro, Arusha, Mwananyamala, Coastal and Tanga hospitals (Fig. 1). Together, these represent 20% of the regional hospitals in Tanzania: regional hospitals are expected to provide specialist level management (including trauma care), and receive referrals from all districts within the catchment area. The median bed capacity of these hospitals during the study time was 440 (range: 295–500), and Arusha (500 beds) had both the highest bed capacity and number of EU beds compared to other hospitals (Table 1). These hospitals were purposively selected, as they are representing the variety of emergency care settings for regional hospitals in Tanzanian, and see a high volume of emergency cases.

**Table 1:** Hospital Characteristics and Patient volume

Regional Hospitals	Overall ***N*** = 2891	Mwananyamala ***N*** = 764	Coastal ***N*** = 555	Tanga ***N*** = 211	Arusha ***N*** = 950	Morogoro ***N*** = 411
Hospital characteristics						
**Patient volume**						
Total patients in EU	31,013	7678	5656	4345	8767	4567
Trauma cases: n (%)	2891 (9.3)	764 (10.0)	555 (9.8)	211 (4.9)	950 (10.8)	411 (9.0)
Gender: Male (%)	2891 (70.7)	764 (74.3)	555 (78.6)	211 (86.7)	950 (63.7)	441 (57.4)
**Infrastructure**	Median (Range)	n	n	n	n	n
Total hospital beds	440 (295–500)	295	210	440	500	450
Emergency Unit beds	4 (2–10)	2	4	4	10	3
Operating theatre	2 (2–3)	2	2	2	3	2
Intensive care beds	1 (0–4)	0	4	0	1	3
X-Ray	1	1	1	1	1	1
CT Scan	0	0	0	0	0	0
Ultrasound	1 (1–3)	1	1	1	3	1
**Staffing resources**						
Clinicians (*N* = 47)	n (%)	n	n	n	n	n
Trauma Surgeon	9 (19.1)	1	2	3	2	1
General Surgeon	9 (19.1)	2	2	2	1	2
Medical Doctors	22 (46.8)	5	3	7	3	4
Assistant Medical Officer	7 (14.9)	1	0	2	1	3
Nurses^a^ (*N* = 69)	n (%)					
Registered Nurses	25 (36.2)	6	3	4	6	6
Enrolled Nurses	17 (24.6)	3	4	2	4	4
Health Attendants	27 (39.1)	5	5	4	8	5
Emergency Unit coverage	Median (Range)					
Clinician per day shift	2 (2–4)	2	2	4	2	3
Clinician per night shift	1	1	1	1	1	1
Nurses per day	4 (2–7)	3	4	2	7	4
Nurses per shift	2 (1–4)	3	2	2	4	1

v^a^Providers allocated to provide care at Emergency Units

**Coastal Regional Hospital** is located in the Coastal region, in eastern Tanzania, positioned along the busiest road connecting the north and south of Tanzania. Hospital caters a catchment area of 1.2 million people.

**Morogoro Regional Referral Hospital** is the regional referral hospital for the Morogoro region, which is about 200 km from Dar es salaam city. Hospital serves a catchment area of 2.3 million people.

**Arusha Regional Hospital**, also known as Mt. Meru Regional Hospital is the regional hospital of Arusha region, located in the north of Tanzania. The hospital has a catchment area of approximately 1.7 million people.

**Tanga Regional Hospital** is the regional referral hospital for the Tanga Region, located in North-Eastern Tanzania along the Indian Ocean. The hospital has a catchment area of 2.0 million people.

**Mwananyamala Regional Hospital** is the designated regional hospital for the Kinondoni administrative region, within Dar es salaam city. The hospital serves a catchment area of 1.1 million people.

These five hospitals are in various stages of the development of emergency and trauma care in Tanzania. During the time of the study, Arusha and Tanga Regional hospital’s EU were undergoing structural renovation and equipment improvement to support enhanced care processes over the next three years. Morogoro and Coastal Regional hospitals are along a section of the Dar es Salaam to Morogoro corridor along which a pre-hospital emergency care pilot study/program will be implemented. Mwananyamala hospital does not have any planned emergency care system improvement.

### Study population

All trauma patients (adult and paediatric) presenting to the study EUs for whom care was documented by a treating provider (medical doctor, Assistant Medical Officer or Clinical Officer).

### Study protocol

This study was conducted from February to July 2018. Trained research assistants - clinical officers (middle level providers with clinical medicine) and diploma nurses - observed in real time the provision of clinical care in the EU for every consecutive patient, and documented all the performed assessment, clinical interventions, and final disposition. In each EU we recruited and trained a total of 3-research assistant to allow 24/7 coverage. After clinicians completed their documentation, research assistants used a purposefully designed case report form (CRF) incorporating the WHO DSI to perform data abstraction from the clinician clinical chart. The research assistants audited the clinical documentation to determine which fields in the WHO DSI were documented by the clinicians, as documented, not documented or incorrectly documented. Furthermore, the research assistants reviewed Ministry of Health issued Facility Register book that is specific for outpatients department (OPD) record (*Health Management Information System (HMIS) book number 5-OPD Register*) and recorded the documentation of variables for each patient in the book. The HMIS book number 5 is a designated book that the Ministry of Heath captures all the disease burden and demographics of patients who presents to the OPD (which includes the EU and designated acute intake areas). At each site, a second (separate) research assistant, independently reviewed a randomly selected subset of 15% of the charts and CRFs, to assess the degree of inter-observer agreement. Lastly, the research assistant used a structured survey tool to interview the administrative providers in each hospital on the human, equipment and structural infrastructure that are relevant for provision of emergency care.

### Data analysis

Data from hand-written CRFs were transferred to an online data capture software (REDCap version 7.2.2, Vanderbilt, Nashville, TN, USA) and then exported to Statistical Package for Social Science (SPSS version 22.0, IBM, Ltd., Carolina, USA) for analysis. Procedure frequency and univariate functions was performed to check for any outliers and clean the dataset. The capture rate of each variable within the WHO DSI was calculated as the number of recommended WHO variables calculated for each patient divided by the total recommended number. A summary proportion of rate of capture for each of the variables with 95% CI’s was calculated for each hospital and measured collectively across all sites. The descriptive statistics of total patients and trauma cases seen in each EU was summarised by frequency distribution tables of proportions for each variable, and median and range were calculated for overall availability of infrastructure and EU coverage. In effort to gain an understanding of whether the registry should be limited to patients with potentially severe/major trauma, we performed a subgroup analysis of rate completeness of WHO recommended documentation for patients who were admitted for inpatient care, died at EU or were transfer to higher level of care. The Inter-observer agreement for the presence and accuracy of trauma injury variable documentation between treating providers was measured by the weighted Cohen’s kappa.

## Results

### Hospital characteristics and patient volume

A total of 31,013 patients were seen in all five Regional hospitals during the study period, of which 2891 (9.3%) presented with trauma-related complaints. The proportion of trauma patients ranged from 4.9 to 10%. Tanga and Mwananyamala had no ICU capacity, and none of the hospitals had a CT scan. Ultrasound was available in each of hospitals at the imaging centre. None of the hospitals had an emergency physician, while in each hospital there were 1 to 3 trauma surgeons, and 3 to 7 medical doctors. Overall, Coastal (3 Registered nurses) had fewer registered nurses compared to the rest of Regional hospitals. On average the night coverage of EUs was 1 clinician, and 2 (Range 1–4) nurses. Table 1.

### Documentation of variables of WHO data set for injury

Overall, the capture rate of all variables was 33.6%. (Table 2**)** Mwananyamala Regional Hospital had the lowest (29.9%) while Morogoro Regional hospital had the highest (36.4%) capture rate. Documentation was most complete for demographics (71.6%), while the initial clinical condition and details of injury were documented in 20.5 and 20.8%, of the total variables respectively and GCS (Glasgow Coma Scale) / AVPU (Alert, Verbal, Pain and Unconscious) was captured in just 3.1% of cases. There was no documentation for the care prior to EU arrival at any hospital; the number of defined serious injuries by the clinical provider’s gestalt was recorded in only 1.3% of cases. The capture rate (33.2%) of variables for sub-group of patient (admitted, died at EU or transferred) was slightly lower than the overall capture rate for all patients (Sup.1).

**Table 2:** Completeness of WHO recommended documentation on clinical chart

Regional Hospitals	Overall ***N*** = 2891	Mwananyamala ***N*** = 764	Coastal ***N*** = 555	Tanga ***N*** = 211	Arusha ***N*** = 950	Morogoro ***N*** = 411
Variable						
**Patient Demographics**	%	%	%	%	%	%
Name of the patient	99.3	99.7	98.6	99.1	99.1	100.0
Age or date of birth	82.0	98.0	75.5	91.0	66.9	91.2
Gender	69.7	73.4	76.8	86.7	62.6	60.6
Address of the patient	83.8	98.7	82.3	83.9	67.8	91.5
Injury Geographical location	14.1	25.4	11.7	11.4	11.2	4.6
Date of EU care	80.9	74.6	82.2	76.8	80.7	93.7
**Initial clinical condition**						
Referral status	8.3	1.8	11.4	23.2	5.6	15.1
UE arrival mode	23.6	0.7	18.6	11.4	50.8	16.5
Signs of life	31.2	0.7	9.9	4.7	73.8	31.9
Time of first vital signs	32.2	40.8	32.1	39.3	15.7	50.6
Initial Heart rate	24.5	1.8	31.4	32.2	31.6	37.2
Initial SBP	18.7	1.4	18.4	18.3	27.9	29.9
Respiratory rate	18.0	1.3	17.3	16.8	27.5	28.5
Saturation of oxygen	13.1	0.1	8.6	9.1	23.1	21.9
Initial GCS/AVPU	3.1	0.1	10.6	8.7	0.6	1.5
First provider assessment time	32.2	40.8	32.1	39.3	15.7	50.6
**Details of injury**						
Mechanism of injury	45.0	44.8	42.3	50.7	46.9	41.8
Mass casualty event	0.5	0.0	1.3	0.5	0.0	1.7
Injury event date	52.2	46.0	58.0	53.6	54.9	49.0
Injury settings	5.3	5.9	4.3	2.4	6.0	5.4
Activity at time of injury	3.3	2.5	2.3	2.4	4.0	3.3
Injury intent	6.8	3.9	7.4	4.3	8.3	9.5
Protective Devices	32.0	31.8	38.2	23.2	30.6	31.9
**Injury Examinatio**						
Type of injury	72.1	61.0	80.7	95.2	67.8	78.8
Injury anatomical location	9.2	3.1	17.7	17.1	9.4	4.4
Defined Serious Injuries	1.3	1.3	1.6	1.4	0.6	2.2
**Emergency Unit details**						
Interventions done at EU	33.0	30.2	22.4	64.9	34.8	31.8
Time of EU departure	15.3	14.8	21.1	15.2	13.7	11.9
EU disposition	62.9	61.9	77.1	59.2	58.0	58.4

Completeness of Ministry of Health required documentation on facility HMIS.

Overall, 1430 (49.5%) of all trauma-related visits seen were documented in the HMIS book by the treating clinicians. (Table 3) Mwananyamala had the highest (57.6%) and Tanga the lowest rate (39.3%) of documentation in the HMIS book. Among the cases reported in the HMIS book, the date of EU care was correctly documented in 77% cases, age 43.6%, diagnosis 66.7%, and outcome in 38.9% cases.

**Table 3:** Completeness of Ministry of Health required documentation on facility HMIS

Regional Hospitals	Overall	Mwananyamala	Coastal	Tanga	Arusha	Morogoro
Total patients in EU	2891	764	555	211	950	411
Patients recorded in HMIS	1430 (49.5)	440 (57.6)	232 (41.8)	83 (39.3)	486 (51.2)	189 (46.0)
**Patients in HMIS**	***N*** **= 1430**	***N*** **= 440**	***N*** **= 232**	***N*** **= 83**	***N*** **= 486**	***N*** **= 189**
**Trauma variables in HMIS**	**%**	**%**	**%**	**%**	**%**	**%**
Date of EC Care	77.0	77.3	74.1	75.9	77.0	80.4
Registration number	39.4	34.1	38.4	50.6	43.2	28.1
Name of the patient	84.0	86.6	80.2	83.2	84.1	82.5
Address of the patient	48.7	48.0	46.6	51.8	52.1	43.4
Age	43.6	45.2	43.5	42.2	41.8	45.5
Sex	79.5	79.5	75.0	75.9	81.1	82.5
Weight	19.3	19.3	20.3	20.5	18.1	20.6
Height	11.7	10.9	7.8	12.0	16.9	4.8
Investigations	46.7	48.2	47.0	55.4	43.0	48.7
Diagnosis	66.7	68.9	62.1	51.8	74.1	55.0
Treatment	56.2	55.7	56.9	41.0	63.8	43.4
Outcome	38.9	36.1	34.5	43.4	42.4	39.7
Remarks	10.8	10.0	10.8	6.0	14.6	5.3

### Performance of interventions and rate of respective documentation

Among the observed procedures, 28.7% of charts documented the assessment of initial clinical condition. Assessment of SBP was the least documented in (20.5%) of charts, while Ultrasound (13.2%) was the least recorded investigation done in EU. Most of patients discharged home (96.0%) were not documented, while most of deceased (96.6%) were recorded in the clinical charts. Table 4.

**Table 4:** Performance of interventions and rate of respective documentation

Variable	Performed	Documented
**Initial clinical condition**	**N**	**n (%)**
Assesment of signs of life	2891	902 (31.2)
Heart rate measurement	2553	708 (27.7)
Measurement of SBP^a^	2626	539 (20.5)
Respiratory rate	1875	519 (27.7)
Saturation of oxygen	657	377 (57.4)
**Interventions done at EU**		
Fracture Reduction	11	4 (36.4)
Fracture splinting	320	308 (96.3)
Open wounds repair	29	21 (72.4)
IVF administration^b^	1708	570 (33.4)
Antibiotic administration	397	28 (7.1)
Analgesic administration	1983	1525 (76.9)
Blood transfusion	39	20 (51.3)
T.T Administration	781	218 (27.9)
Chest tube placement	125	125 (100)
Foreign body removal	12	1 (8.3)
**Investigations done**		
Chest X-ray	447	261 (58.4)
Long bone X-ray	754	420 (55.7)
Ultrasound	257	34 (13.2)
**Disposition**		
Referred to higher facilities	537	514 (95.7)
Admitted to inpatient service	1248	1122 (89.9)
Died in EU	148	143 (96.6)
Discharged home	958	38 (4.0)

*SBP-Systolic Blood Pressure, **IVF-Intravenous Fluid

## Discussion

Our manuscript describes a comprehensive assessment of the baseline capture rate of WHO-defined injury variables in a Sub Saharan country. In this study, we found a substantial lack of documentation of trauma variables in both general trauma patients as well as a sub-group of patients with potentially severe or major injuries. There is paucity of studies in this field, but some available studies have demonstrated the usability of implemented TRs without evaluating the baseline capture rate of variables [7, 20]. The development of successful national TRs is dependent on availability of standard set of injury variables that are consistently collected from all trauma patients [7, 21, 22]. In all facilities, we also found significant under-documentation of patients in the facility HMIS register that is used to submit the facility trauma data to the Ministry of Health.

Many attempts to develop TRs in sub Saharan Africa have largely been unsuccessful or unsustainable beyond the initial research phase for a number of reasons, including lack of uniform clinical documentation of injury variables [23–26]. The HMIS register is currently the only tool that provides injury data to inform the Ministry of Health about the national burden of injury, and so the quality and consistency of the data gathered is of paramount importance [27]. However, while we recorded a very low rate of variables documented in the facility HMIS register, we also note that even at full capture the variables will not serve the needs of a TR, given that it records less than one fifth of WHO DSI variables.

Despite an overall low capture rate of injury variables, we found a high rate of documentation for patients demographics, which is attributed to the fact that most of the patients have to be registered for billing purposes [28]. The initial clinical presentation of the patient was not documented for over three quarter of variables needed to inform the TR. Conscious level is an important marker that informs both care pathways and clinical prognosis: despite this importance, GCS or AVPU was almost never recorded [29–31].

The documentation of pre hospital care and mechanism of injury variables can inform TRs and help to demonstrate the priority areas for strengthening care to optimize outcome [3]. Care prior to EU, signs of life, and mechanism of injury were documented in less than half of patients, and in some data providers mixed the chief complaint and mechanisms of injuries, limiting potential usability of data gathered to inform on the status of recommended injury prevention interventions. Training of providers on importance of injury variables can improve understanding and improve documentation [32].

Lack of resources and increased patient workload are known to impact documentation of vital signs in different settings [33]. Vital signs were documented in less than one fifth of patients, with saturation of oxygen being recorded in the lowest proportion of patients. Only two hospitals had either a cardiac monitor or a pulse oximeter, obviously contributing towards the low oxygen saturation capture rate. In the current reality of the emergency care system, it will be very difficult to document severity of injuries seen in Tanzania [34].

We observed that a substantial proportion of performed assessment and interventions were not documented in the clinical chart. Surgical interventions, for example chest tube placement, was documented more thoroughly, and we believe this is related to both billing and administrative needs of documentation of surgical procedures [27]. In all EUs, most discharged patients do not receive any documentation; we observed most patients receiving verbal discharge with follow up dates. We believe this is related to the lack of standardised EU documentation, and lack of EU filing system for patients who are not admitted. In facilities with the filing system the patients are provided with a copy of the discharge instructions that are filed with the hospital registry.

We noted a substantial shortage of EU beds with an average of one bed for every 43 patients in all EUs, giving ratio of one bed to four trauma patients. Compounding the challenge of EU bed capacity is the shortage of ICU bed in all the regional hospitals; two regional hospitals had no ICU capacity at all. In all EUs we found a low staffing level, similar to that observed in previous studies of EU staffing in Tanzania [9]. A combination of limited infrastructure, low staffing and shortage of supplies might further explain the observed low capture rate of injury variables.

### Limitations

The study was conducted in five regional hospitals with variable resources, patient flow system and volume. This may not reflect the reality in the rest of the health facilities in Tanzania. Our utilization of trauma form with complete WHO data set to assess the capture rate of variables in all patients regardless of the severity of injury might have contributed to poor documentation of and a low capture rate given that providers might not document variables they feel irrelevant for minor trauma, however we noted the same poor documentation for a sub-group of trauma patients with likelihood of serious or major injuries (admitted, died in EU and referred), as well as for variables that are part of core WHO data set, meant to be documented for any patient.

## Conclusion

In regional hospitals of Tanzania, there is inadequate documentation of the minimum trauma variables specified in the WHO DSI. Reasons for this were not explored in this study, but will need to be addressed in order to improve documentation to inform a national injury registry.

## Supplementary information

**Additional file 1: Sup 1.** Completeness of WHO recommended documentation for patients who were admitted, died or transferred to higher-level facility.

## Data Availability

The datasets used and/or analysed during the current study are available from the corresponding author on reasonable request.

## Abbreviations

CRF: Case Report Form
DSI: Data set for injury
EU: Emergency Unit
HIC: High Income Country
HMIS: Health Management Information System
ICU: Intensive Care Unit
OPD: Out patient department
WHO: World Health Organisation

## References

[1] Zehtabchi S, Nishijima DK, McKay MP, Clay Mann N (2011). Trauma Registries: History, Logistics, Limitations, and Contributions to Emergency Medicine Research. Acad Emerg Med.

[2] The Goals, Development, and Use of Trauma Registries and Trauma Data Sources in Decision Making in Injury. PubMed Journals. Available from: https://ncbi.nlm.nih.gov/labs/articles/7900000/. [cited 2017 Jun 26]. 10.1016/s0039-6109(16)46590-4 7900000

[3] Nwomeh BC, Lowell W, Kable R, Haley K, Ameh EA (2006). History and development of trauma registry: lessons from developed to developing countries. World J Emerg Surg.

[4] Kobusingye O, Guwatudde D, Owor G, Lett R (2002). Citywide trauma experience in Kampala, Uganda: a call for intervention. Inj Prev.

[5] Kobusingye OC, Lett RR (2000). Hospital-based trauma registries in Uganda. J Trauma.

[6] Taye M, Munie T (2003). Trauma registry in Tikur Anbessa Hospital, Addis Ababa. Ethiopia Ethiop Med J.

[7] Mehmood A, Razzak JA, Kabir S, MacKenzie EJ, Hyder AA (2013). Development and pilot implementation of a locally developed Trauma Registry: lessons learnt in a low-income country. BMC Emerg Med.

[8] Manzi F, Schellenberg JA, Hutton G, Wyss K, Mbuya C, Shirima K (2012). Human resources for health care delivery in Tanzania: a multifaceted problem. Hum Resour Health.

[9] Sirili N, Kiwara A, Gasto F, Goicolea I, Hurtig A-K. Training and deployment of medical doctors in Tanzania post-1990s health sector reforms: assessing the achievements. Human Resources Health. 2017;15(1). 10.1186/s12960-017-0202-7. 10.1186/s12960-017-0202-7 PMC5381067 28376823

[10] Nicks BA, Sawe HR, Juma AM, Reynolds TA (2012). The state of emergency medicine in the United Republic of Tanzania. African J Emergency Med.

[11] Boniface R, Museru L, Kiloloma O, Munthali V (2016). Factors associated with road traffic injuries in Tanzania. Pan Afr Med J.

[12] Chalya PL, Dass RM, McHembe MD, Mbelenge N, Ngayomela IH, Chandika AB (2013). Citywide trauma experience in Mwanza, Tanzania: a need for urgent intervention. J Trauma Manag Outcomes.

[13] Chalya PL, Mabula JB, Ngayomela IH, Kanumba ES, Chandika AB, Giiti G (2010). Motorcycle injuries as an emerging public health problem in Mwanza City, North-Western Tanzania. Tanzan J Health Res.

[14] WHO (2020). Emergency and trauma care.

[15] WHO (2020). WHO International Registry for Trauma and Emergency Care. WHO International Registry for Trauma and Emergency Care.

[16] World Bank (2017). World bank data. World bank data.

[17] Reynolds TA, Mfinanga JA, Sawe HR, Runyon MS, Mwafongo V (2012). Emergency care capacity in Africa: A clinical and educational initiative in Tanzania. J Public Health Policy.

[18] Baker T, Lugazia E, Eriksen J, Mwafongo V, Irestedt L, Konrad D (2013). Emergency and critical care services in Tanzania: a survey of ten hospitals. BMC Health Serv Res.

[19] Koka PM, Sawe HR, Mbaya KR, Kilindimo SS, Mfinanga JA, Mwafongo VG (2018). Disaster preparedness and response capacity of regional hospitals in Tanzania: a descriptive cross-sectional study. BMC Health Services Res.

[20] Chichom-Mefire A, Nwanna-Nzewunwa OC, Siysi VV, Feldhaus I, Dicker R, Juillard C (2017). Key findings from a prospective trauma registry at a regional hospital in Southwest Cameroon. PLoS One.

[21] Mehmood A, Chan E, Allen K, Al-Kashmiri A, Al-Busaidi A, Al-Abri J, et al. Development of an mHealth trauma registry in the Middle East using an implementation science framework. Glob Health Action. 2017;10(1) Available from: https://www.ncbi.nlm.nih.gov/pmc/articles/PMC5678440/. [cited 2019 Dec 12]. 10.1080/16549716.2017.1380360 PMC5678440 29027507

[22] Juillard CJ, Stevens KA, Monono ME, Mballa GAE, Ngamby MK, McGreevy J (2014). Analysis of prospective trauma registry data in francophone Africa: a pilot study from Cameroon. World J Surg.

[23] Adeloye D, Thompson JY, Akanbi MA, Azuh D, Samuel V, Omoregbe N (2016). The burden of road traffic crashes, injuries and deaths in Africa: a systematic review and meta-analysis. Bull World Health Organ.

[24] Boniface R, Museru L, Kiloloma O, Munthali V (2016). Factors associated with road traffic injuries in Tanzania. Pan Afr Med J.

[25] Chalya PL, Mabula JB, Dass RM, Mbelenge N, Ngayomela IH, Chandika AB (2012). Injury characteristics and outcome of road traffic crash victims at Bugando medical Centre in Northwestern Tanzania. J Trauma Manag Outcomes..

[26] Chokotho LC, Mulwafu W, Nyirenda M, Mbomuwa FJ, Pandit HG, Le G (2019). Establishment of trauma registry at Queen Elizabeth Central Hospital (QECH), Blantyre, Malawi and mapping of high risk geographic areas for trauma. World J Emerg Med.

[27] Ministry of Health (2017). Tanzania HMIS. Tanzania Health Management Information System.

[28] Peck R, Mghamba J, Vanobberghen F, Kavishe B, Rugarabamu V, Smeeth L (2014). Preparedness of Tanzanian health facilities for outpatient primary care of hypertension and diabetes: a cross-sectional survey. Lancet Glob Health.

[29] Jain S, Teasdale GM, Iverson LM (2019). Glasgow Coma Scale. StatPearls.

[30] Sternbach GL (2000). The Glasgow coma scale. J Emerg Med.

[31] Cicero MX, Cross KP (2013). Predictive value of initial Glasgow coma scale score in pediatric trauma patients. Pediatr Emerg Care.

[32] Lorenzetti DL, Quan H, Lucyk K, Cunningham C, Hennessy D, Jiang J, et al. Strategies for improving physician documentation in the emergency department: a systematic review. BMC Emerg Med. 2018;18 Available from: https://www.ncbi.nlm.nih.gov/pmc/articles/PMC6297955/. [cited 2019 Dec 12]. 10.1186/s12873-018-0188-z PMC6297955 30558573

[33] Recio-Saucedo A, Maruotti A, Griffiths P, Smith GB, Meredith P, Westwood G (2018). Relationships between healthcare staff characteristics and the conduct of vital signs observations at night: Results of a survey and factor analysis. Nurs Open.

[34] Javali RH, Krishnamoorthy PA, Srinivasarangan M, Suraj S (2019). Comparison of Injury Severity Score, New Injury Severity Score, Revised Trauma Score and Trauma and Injury Severity Score for Mortality Prediction in Elderly Trauma Patients. Indian J Crit Care Med.

