# Supplementary material for: Trauma care and capture rate of variables of World Health Organisation data set for injury at regional hospitals in Tanzania: first steps to a national trauma registry
Source: BMC Emerg Med. 2020 Apr 23;20:29. doi: 10.1186/s12873-020-00325-y (PMC7178583; doi:10.1186/s12873-020-00325-y)
Supplement: Supplementary file 1 — Additional file 1: Sup 1. Completeness of WHO recommended documentation for patients who were admitted, died or transferred to higher-level facility. [file 12873_2020_325_MOESM1_ESM.docx]

| **Regional Hospitals** | **Overall** | **Mwananyamala** | **Coastal** | **Tanga** | **Arusha** | **Morogoro** |
| --- | --- | --- | --- | --- | --- | --- |
| **Variable** | **N=1933** | **N=547** | **N=374** | **N=177** | **N=534** | **N=301** |
| **Patient Demographics** | % | % | % | % | % | % |
| Name of the patient | 99.4 | 99.8 | 98.9 | 98.9 | 99.3 | 100 |
| Age or date of birth | 83.3 | 97.4 | 75.1 | 90.4 | 67.6 | 91.7 |
| Gender | 70.2 | 73.9 | 75.9 | 88.1 | 62.0 | 60.5 |
| Address of the patient | 83.9 | 98.5 | 82.1 | 82.5 | 66.3 | 91.4 |
| Injury Geographical location | 8.5 | 18.6 | 5.1 | 12.4 | 2.4 | 2.7 |
| Date of EU care | 86.0 | 81.4 | 88.2 | 75.1 | 87.8 | 95.0 |
| **Initial clinical condition** |  |  |  |  |  |  |
| Referral status | 10.0 | 1.8 | 14.4 | 21.5 | 5.8 | 20.3 |
| UE arrival mode | 22.7 | 0.7 | 20.1 | 12.4 | 54.7 | 15.3 |
| Signs of life | 31.2 | 0.5 | 8.6 | 5.1 | 85.6 | 34.2 |
| Time of first vital signs | 26.0 | 32.7 | 26.7 | 39.0 | 3.4 | 45.2 |
| Initial Heart rate | 24.2 | 1.6 | 30.7 | 31.6 | 32.8 | 37.9 |
| Initial SBP | 18.4 | 1.1 | 19.3 | 18.4 | 28.7 | 30.6 |
| Respiratory rate | 17.6 | 1.1 | 17.9 | 16.7 | 28.1 | 28.9 |
| Saturation of oxygen | 13.0 | 0.2 | 8.0 | 9.2 | 24.9 | 23.3 |
| Initial GCS/AVPU | 4.1 | 0.2 | 13.4 | 9.2 | 1.1 | 2.0 |
| First provider assessment time | 26.0 | 32.7 | 26.7 | 39.0 | 3.4 | 45.2 |
| **Details of injury** |  |  |  |  |  |  |
| Mechanism of injury | 51.4 | 53.9 | 57.2 | 48.0 | 43.6 | 55.5 |
| Mass casualty event | 0.5 | 0.0 | 1.9 | 0.6 | 0.0 | 0.7 |
| Injury event date | 50.5 | 48.0 | 55.9 | 49.7 | 52.1 | 46.0 |
| Injury settings | 6.3 | 6.2 | 5.6 | 2.3 | 8.4 | 5.6 |
| Activity at time of injury | 3.7 | 2.6 | 1.9 | 2.8 | 5.6 | 5.0 |
| Injury intent | 7.9 | 4.4 | 8.6 | 5.1 | 11.0 | 9.6 |
| Protective Devices | 29.5 | 30.7 | 37.4 | 26.6 | 23.6 | 29.9 |
| **Injury Examination** |  |  |  |  |  |  |
| Type of injury | 69.7 | 71.8 | 77.5 | 62.7 | 68.1 | 63.1 |
| Injury anatomical location | 9.7 | 3.3 | 16.6 | 18.1 | 11.2 | 5.0 |
| Defined Serious Injuries | 1.2 | 1.1 | 1.6 | 1.1 | 0.4 | 2.4 |
| **Emergency Unit details** |  |  |  |  |  |  |
| Interventions done at EU | 35.1 | 37.8 | 25.0 | 59.9 | 31.3 | 34.8 |
| Time of EU departure | 13.0 | 13.7 | 18.4 | 12.4 | 10.5 | 10.0 |
| EU disposition | 60.4 | 63.8 | 79.7 | 52.0 | 47.4 | 58.5 |
